# How to Improve Clinical Outcomes in Patients with Tachycardia-Induced Cardiomyopathy. Comment on Katz et al. Long-Term Outcomes of Tachycardia-Induced Cardiomyopathy Compared with Idiopathic Dilated Cardiomyopathy. *J. Clin. Med.* 2023, *12*, 1412

**DOI:** 10.3390/jcm12155065

**Published:** 2023-08-01

**Authors:** Naoya Kataoka, Teruhiko Imamura

**Affiliations:** Second Department of Internal Medicine, University of Toyama, Toyama 930-0194, Japan

The clinical course of tachycardia-induced cardiomyopathy (TICM) has not yet been well studied thus far. Katz and colleagues showed that patients with TICM had a statistically comparable prognosis to those with idiopathic dilated cardiomyopathy (IDCM) [1]. Several concerns have been raised to strengthen their findings and deepen this discussion.

The definitions of each disease (i.e., TICM and IDCM) may be unclear. In their study, the patients who achieved an improvement in the left ventricular ejection fraction (LVEF) >15% due to rate control or rhythm control were assigned to the TICM group [1]. Here, four patients received cardiac resynchronization therapy, which often improves cardiac function by improving cardiac dissynchrony. Such patients may not have TICM. In the IDCM group, patients with tachycardia were excluded [1]. However, five patients with an atrial fibrillation or atrial flutter were also included in this group. These arrhythmias, even if they are paroxysmal ones, can have a negative impact on the LVEF. Not all asymptomatic tachycardia cases can be detected without cardiovascular implantable electronic devices. We propose the definition of TICM patients as those with an improvement in LV systolic function following the elimination of the arrhythmias without using any devices that affect ventricular function [2].

Another concern is a therapeutic strategy used for those with an impaired LVEF and atrial fibrillation. In their study, 22% of the TICM patients died during a 6-year observation period, despite a median improvement in the LVEF of up to 55% [1]. This mortality rate is higher than those in other previous large-scale studies [3]. Could the authors explain this discrepancy? One explanation could be a difference in the therapeutic strategy for patients with an impaired LVEF and atrial fibrillation. In the current era following the prospective randomized trial entitled Catheter Ablation for Atrial Fibrillation with Heart Failure (CASTLE-AF) [3], catheter ablation is highly recommended for such clinical situations [4,5]. Medical intervention to control rate/rhythm does not have a positive prognostic impact in this cohort [6]. However, only 20% of their cohort received catheter ablation. Catheter ablation should also reduce the incidence of recurrent arrhythmias that require re-hospitalization.

Finally, guideline-directed medical therapy, such as the fantastic four, is highly recommended for patients with heart failure with a reduced LVEF [7]. Could the authors clarify the medication list, including mineralocorticoid receptor antagonists and sodium-coupled glucose transporter 2 inhibitors? Beta blockers were prescribed in 35% of the patients, and renin-angiotensin system inhibitors were prescribed in 27% [1].
